# Validity of Pleuroscopy in Evaluating Pleural Effusions of Uncertain Etiology at the Cardiothoracic Unit of Minia University Hospital, Egypt

**DOI:** 10.7759/cureus.59300

**Published:** 2024-04-29

**Authors:** Ali Abdelaziz, Rofida Hassan, Rasha Abdelfattah, Ali Hassan, Hager Yehia, Ahmed Mady, Elham Abdelhady

**Affiliations:** 1 Department of Chest Diseases, Minia University, Minia, EGY; 2 Department of Chest Diseases, Assiut University, Assiut, EGY

**Keywords:** malignant effusion, empyema, diagnostic yield, exudative pleural effusion, medical thoracoscopy

## Abstract

Introduction: Pleural effusion is a medical condition where an excessive amount of fluid accumulates in the pleural space. This can be caused by inflammation or malignant growth in the body. Doctors use medical thoracoscopy for both diagnostic and therapeutic purposes. This technique allows them to view the internal pleural surfaces and take biopsies of any abnormal lesions within the pleural cavity.

Objective: This work aimed to evaluate the diagnostic value of pleuroscopy in patients with undiagnosed exudative pleural effusion.

Patients and methods: A study was conducted on 61 patients who had undiagnosed exudative pleural effusion and were admitted to the chest department at the cardiothoracic unit of the Minia University Hospital. All patients provided written consent and underwent a complete history and clinical examination. Standard laboratory tests, including routine liver and kidney function tests, a complete blood count, and a coagulation profile, were conducted on all patients, along with chest X-rays. If necessary, a chest CT scan was also performed. Diagnostic thoracentesis was done, and the pleural fluid was analyzed for sugar, protein, and lactate dehydrogenase and sent for bacteriological analysis (Gram stain, culture, and acid-fast bacilli smear) and cytopathological examination. Medical thoracoscopy was performed in cases where an etiological diagnosis was not established.

Results: A total of 61 patients with undiagnosed exudative pleural effusions were included. A definitive etiological diagnosis was reached in 58 (95%) patients. In 47 (77%) of the studied group, malignant etiology was confirmed; nine (14.8%) had tuberculous pleurisy, one (1.6%) had empyema, and one (1.6%) had inflammatory/autoimmune pleurisy. A definite diagnosis was not reached in three (5%) patients. The malignant pathology was caused by bronchogenic carcinoma in 20 (42.5%) cases, malignant mesothelioma in 10 (21.3%) cases, metastatic malignant deposits from other organs in six (12.7%) cases, and lymphoma in three (6.5%) cases. No serious adverse events related to the procedure were recorded. The most common minor complications were transient chest pain in 34 (55.7%) patients, followed by surgical emphysema in 10 (16.4%) patients.

Conclusion: Pleuroscopy is an effective diagnostic tool for identifying the cause of pleural effusion when it is unclear. It is a minimally invasive and straightforward procedure associated with high diagnostic accuracy and low complication rates.

## Introduction

A pleural effusion is an abnormal accumulation of fluid in the pleural space. The imbalance between the formation and reabsorption of pleural fluid is the underlying pathophysiological mechanism of pleural effusion. A myriad of diseases can cause pleural effusion. The pathogenic mechanisms for the formation of pleural effusion include either an increase of the hydrostatic pressure gradient that causes transudation of fluid or increased extravasation of fluid from the pleural vessels which is termed exudation [1]. Establishing the etiology of the pleural effusion is sometimes a challenging process that necessitates numerous investigations. The diagnostic workup of a case of pleural effusion usually starts with careful history taking and physical examination. The initial investigations usually start with a chest X-ray. Establishing the underlying cause often requires invasive procedures, from thoracentesis to percutaneous pleural biopsy and thoracoscopy [2]. The clinical application of thoracoscopy provides an excellent diagnostic method for undiagnosed pleural effusions, improving the positive diagnostic rate of pleural diseases when thoracocentesis and closed pleural biopsy are non-diagnostic [3]. The study aims to evaluate the safety and efficacy of medical thoracoscopic pleural biopsy in patients with undiagnosed exudative pleural effusion.

## Materials and methods

This is a descriptive cross-sectional study including 61 patients with exudative, undiagnosed pleural effusion. The patients were admitted to the chest department of the cardiothoracic unit at Minia University Hospital between June 2021 and January 2023. This study was approved by the Research Ethics Committee of the Faculty of Medicine, Minia University (approval no. 12:1/2021). Patients were enrolled in the study after receiving their written informed consent.

Selection criteria

The current study included patients of both genders who presented with an undiagnosed exudative pleural effusion and had consented to participate. Patients who had contraindications for performing thoracoscopy, such as uncorrected coagulation disorder, extensive loculations, or hemodynamically unstable patients, and those who did not consent, were excluded from the study.

All patients underwent a thorough medical history and clinical examination, including an evaluation of blood pressure, pulse rate, and oxygen saturation. They also underwent chest X-rays (posteroanterior and lateral views) and CT scans of the chest with contrast. Additionally, routine laboratory investigations such as complete blood count, liver and kidney function tests, prothrombin time, concentration, and international normalized ratio (INR) were performed for all patients. Diagnostic pleural fluid aspiration was conducted, and the fluid was analyzed chemically (for sugar, total protein, lactate dehydrogenase (LDH), and adenosine deaminase (ADA) if necessary), bacteriologically (through staining for acid-fast bacilli, gram stain, culture, and sensitivity), and cytologically for any abnormal cells.

Thoracoscopic procedure

Patients with exudative pleural effusion inconsistent with infection and with no extrapulmonary cause for effusion were followed directly by rigid medical thoracoscopy to reduce the time needed for diagnosis, the number of patient visits to the hospital, the number of invasive procedures, and the strain on hospital resources during the COVID-19 era. A Karl-Storz rigid thoracoscope with a cold light source was used. In a lateral decubitus position with the affected side up, the patient was often laid down on the healthy side. Entry was done through the 5th or 6th intercostal space with ultrasound guidance. Local anesthesia with intravenous conscious sedation was usually adequate. 

Two to six biopsies were taken from visible lesions in the parietal pleura under direct vision. A lateral ‘lift and peel’ technique was used. In cases where no visible abnormalities were observed in the parietal pleura, multiple biopsies were taken from different areas. The biopsies were sent for both bacteriological and histopathological examinations. A chest tube was inserted after finishing the procedures. 

## Results

In our present study, 61 patients had unexplained exudative pleural effusion over 18 months. The characteristics of the patients and their clinical presentations are summarized in Table 1. The mean age was 58.34 ±16.5 with a range of 22 to 83. Most of them were males (63.9%). The most common symptoms were breathlessness (83.6%), cough (62.3%), chest pain (57.3%), and toxic manifestations (52.2%).

**Table 1:** Demographic data and clinical presentations of the studied patients

Variable	Number (%)
Age (mean ± SD)	58.34± 16.5
Male	39 (63.9%)
Smoking state	
Smoker	28 (45.9%)
Non-smoker	33 (54.1%)
Clinical presentations	
Cough	38 (62.3%)
Shortness of breath	51 (83.6%)
Chest pain	35 (57.3%)
Toxic manifestations	32 (52.2%)
Clubbing	8 (13.1%)
Hemoptysis	2 (3.3%)

As regards the site of effusion, there were 28 (45.9%) right-sided, 26 (42.6%) left-sided, and seven (11.5%) bilateral as shown in Table 2. The amount was moderate in 37.7% of cases and large to massive in 62.3% of cases. The appearance of pleural effusion was bloodstained in 37.7% and yellowish in 62.3% of cases, with predominant lymphocytes in 90%. Atypical cells suggesting malignancy were present in 29.5% of cases. The CT findings included pulmonary consolidation or infiltration (11.5%), pulmonary mass (4.9%), pleural nodularity or mass (11.5%), and fluid encystation (13%).

**Table 2:** Characteristics of pleural effusion and CT findings of the studied patients

Variable	Number (%)
Site of effusion	
Right	28 (45.9%)
Left	26 (42.6%)
Bilateral	7 (11.5%)
Amount of effusion	
Moderate	23 (37.7%)
Large to massive	38 (62.3%)
Pleural fluid color	
Yellowish	38 (62.3%)
Hemorrhagic	23 (37.7%)
Cytological examination	
Lymphocyte	53 (90.2%)
Neutrophils	2 (3.3%)
Hypocellular	4 (6.5%)
Presence of atypical cells in fluid	18 (29.5%)
CT findings	
Pleural nodules/mass	7 (11.5%)
Lung mass	3 (4.9%)
Parenchymal abnormalities	7 (11.5%)
Fluid encystation	8 (13.1%)

When performing medical thoracoscopy, we found pleural nodules or pleural masses in 45 cases (73.8%), and adhesions in 20 (32.8%), as shown in Table 3.

**Table 3:** Thoracoscopic findings among the studied patients

Variables	Number (%)
Pleural nodule/masses	45 (73.8%)
Diffuse pleural thickening	19 (31.1%)
Adhesion	20 (32.8%)
Hyperemia	6 (10%)

A definitive diagnosis was obtained through the thoracoscopic biopsies in 58 (95%) out of 61 patients. In three cases (5%) the biopsies were non-conclusive. Malignancy was confirmed in 47 (77%), and tuberculosis in nine (14.8%) patients, as shown in Table 4.

**Table 4:** Thoracoscopic biopsy result and the diagnostic yield of medical thoracoscoy in the studied group(N=61)

Diagnosis	Number (%)
Malignancy	47 (77%)
Tuberculosis	9 (14.8%)
Empyema	1 (1.6%)
Inflammation/autoimmune	1 (1.6%)
Undiagnosed	3 (4.9%)
Diagnostic yield of medical thoracoscopy in the studied group	
Diagnosed	58 (95%)
Undiagnosed	3 (5%)

The malignant pathology was caused by bronchogenic carcinoma in 20 (42.5%) cases, malignant mesothelioma in 10 (21.3%) cases, metastatic malignant deposits from other organs in six (12.7%) cases, and lymphoma in three (6.5%) cases (Table 5).

**Table 5:** Histopathological types of malignancy among the studied patients (n=47)

Types of malignancy	Number (%)
Metastatic adenocarcinoma (lung)	20 (42.5%)
Mesothelioma	10 (21.3%)
Lymphoma	3 (6.5%)
Poorly differentiated carcinoma	8 (17%)
Metastatic adenocarcinoma (other than lung)	6 (12.7%)

During our study, no serious adverse events were recorded in any patient. There were 34 patients (55.7%) who had pain at the puncture site, 10 (16.4%) had surgical emphysema, five (8.2%) had wound infection, and only one (1.6%) had empyema (Table 6).

**Table 6:** Complications of thoracoscopy in the studied patients (n=61)

Complications	Number (%)
Pain at the puncture site	34 (55.7%)
Surgical emphysema	10 (16.4%)
Empyema	1 (1.6%)
Wound infection	5 (8.2%)
Intraoperative bleeding	0 (0%)

## Discussion

Among patients with undiagnosed pleural effusions, malignant etiology for the effusion is a significant consideration. This necessitates the confirmation of the etiology for pleural effusion through pleural biopsy. Reaching a definitive histological diagnosis and identifying tumor receptor status have an important implication for treatment options and decision-making for both the patient and clinician [4]. Thoracoscopy allows direct visualization of the pleura and large volume of thoracocentesis. Both advantages make thoracoscopy a valuable and important option for patients in whom the cause of the effusion had not been established using other less invasive procedures [5]. In our study, most of the patients were males 39 (63.9%) and this is in agreement with previous studies [6-8]. The main respiratory complaints were dyspnea in 51 patients (83.6%), followed by cough in 38 (62.3%). This was also reported by Patil et al. [9]. We noticed that 28 (45.9%) study patients had right-sided pleural effusion, 26 (42.6%) had left-sided pleural effusion, and seven (11.5%) had bilateral effusion, which was also observed by Yb et al. [4].

In the current study, pleural fluid cytology was positive for atypical cells in 18 (29.5%) cases but was inconclusive, while in the remaining 43 (70.5%) cases it was negative, and this is consistent with Mohamed et al. [10] who found that pleural fluid atypical cells were present only in 30% of malignant cases. As regards CT findings in our study, pleural fluid encystation, pleura nodules/mass, and parenchymal abnormalities were the most common CT findings consistent with Embarak et al.'s [11] study. In the current study, there were eight cases (13.1%) with fluid encystation, seven (11.5%) had pleura nodules/mass, seven (11.5%) had parenchymal abnormalities, and three (4.9%) had lung mass. Embarak et al. [11] reported chest findings that revealed that 34 (37.7%) patients had pleural nodules, 10 (11.1%) showed pleural encystations, and seven (7.8%) showed pulmonary infiltrations.

Regarding thoracoscopic findings, our study found a great portion of patients had pleural nodules, pleural adhesions, and diffuse pleural thickening, which is in agreement with Eaid et al. [12]. In the current study, pleural nodules were found in 45 patients (73.8%), adhesions in 20 patients (32.8%), and diffuse pleural thickening in 19 patients (31.1%). These findings are compatible with Eaid et al. [12], who illustrated that regarding thoracoscopic findings, 104 patients (71.7%) had multiple nodules, 14 patients (9.7%) had pleural adhesions, and eight patients (5.5%) had pleural thickening. In the current study, a definitive diagnosis through thoracoscopic biopsies was obtained in 58 (95%) patients. These results align with other studies in Egypt [13,14], India [6], Iran [15], and Denmark [16], that range from 87% to 97%. Some studies had a lower diagnostic yield of 66.7% and 74.3% in patients with undiagnosed pleural effusions [17,18].

In this study, it was found that malignancy was diagnosed in the majority of patients with undiagnosed pleural effusions. Our thoracoscopic biopsy results revealed that malignancy was diagnosed in 47 (77%) of patients, of which, nine (14.8%) were due to tuberculosis, three (4.9%) were undiagnosed, one (1.6%) was due to empyema, and another (1.6%) due to inflammation/ autoimmune disorder. These results are compatible with a study by Rai et al. [5], who found malignancy in (58/76) 76.31% of patients with unexplained pleural effusion, and Hucker et al. [19] who found malignancy in 59% of cases. Wu Yb et al.'s [4] study found malignancy in only 41.1%; this could be explained by the difference in sample size. In Egypt, many previous studies found that the most common diagnosis was malignancy, as Mohamed and Shaban [13] found malignancy in 87/117 (74.4%) patients, Ahmed et al. [14] found malignancy in 46 (92%) patients, and Halima et al.'s [20] study reported malignancies in 83.53% of patients. Adenocarcinoma of the lung was the most common type of malignancy, causing pleural effusion that was observed in (20/47) 42.5% of patients. This is in agreement with previous studies [9,21]. Malignant pleural mesothelioma (MPM) was diagnosed in (10/47) 21.3 % of patients, while other studies from Egypt such as the one by Mohamed and Shaban [13] revealed MPM in 47.01% of patients, Halima et al., [20] revealed MPM in 49.41% of patients, and Helala et al. [22] revealed MPM in 53.6%. This may be explained by the fact that most of the patients in the three previous studies were working or living in neighborhoods with asbestos factories.

We didn’t record any serious adverse events related to the procedure. The most common minor complication was transient chest pain in 34 (55.7%) patients from the site of the chest tube insertion, followed by surgical emphysema in 10 (16.4%) patients. The absence of serious side effects and the high diagnostic yield of medical thoracoscopy observed in our cohort of patients support its important role in the diagnosis of patients with unexplained pleural effusion and these results are in agreement with data from a meta-analysis conducted by Agarwal et al. [23].

This study is limited by the lack of a comparison group in whom biopsy was taken with ultrasound guidance or CT-guided biopsy. The availability of thoracoscopy, the need to decrease the number of patient visits to the hospital, and the strain on hospital resources during the COVID-19 era were the reasons behind this.

## Conclusions

Undiagnosed exudative pleural effusion is a common medical problem. A pleural biopsy for confirmation of the etiology of undiagnosed pleural effusion is usually required. Medical thoracoscopy is a valuable tool in the diagnostic workup for patients in whom the etiology of the effusion is not reached through other conventional techniques. It is a minimally invasive technique that is associated with high diagnostic yield and non-serious complications at lower rates. The most commonly reported complications were pain at the puncture site and surgical emphysema. 
